# Croton-Chloral Hydrate in Megrim*Read before the Medical Section at the Annual Meeting of the British Medical Association in Norwich, August, 1874.

**Published:** 1875-04

**Authors:** Sydney Ringer

**Affiliations:** Professor of Materia Medica in University College, London


### CROTON-CHLORAL HYDRATE IN MEGRIM.*

BY SYDNEY RINGER, M.D.,

*Professor of Materia Medica in University College, London.*

It is hardly necessary to observe that under the term megrim I include those affections commonly called sick-headache, bilious-headache, nervous sick-headache, and hemicrania. The most characteristic and commonest symptoms of megrim are headache and sickness; but, in a typical case, these symptoms are preceded by other significant and interesting phenomena. At the onset of an attack, a peculiar affection of the sight first occurs, soon to be followed by perversion of the sense of touch and of the muscular sense in the arms and legs; by disordered speech and defective ideation; the headache then comes on, and, as it becomes intensified, nausea gradually sets in.

The affection of the sight may consist of mere absence of vision, beginning at the centre of circumference of the field of sight.—When at the circumference, the defect is generally situate to the right or left of the axis of vision. From the centre of the visual field, the blind spot gradually expands, and as it enlarges it gradually clears up in the centre, and so gradually disappears to the circumference. As the blind spot expands, its margin is often lighted up with spectra variously described as glimmering, dazzling, bright zig-zag lines, coruscations, etc.

In ten minutes to half an hour, on one or both sides of the body, numbness and loss of sensibility occur, followed by tingling, formication, "pins and needles," felt most distinctly in the hands, tongue and lips. Speech is commonly disordered, the aberration in some cases being simply memorial, in others motorial; in others, again, these two derangements of speech are more or less combined. In other words, one patient forgets his words, another forgets how to utter them, whilst a third manifests a combination of these two defects. There is, too, loss of memory, confusion of ideas, and a bewildering feeling, as if the patient were going out of his mind. In half an hour or a little longer, these phenomena are followed

* Read before the Medical Section at the Annual Meeting of the British Medical Association in Norwich, August, 1874.

by headache, which is generally felt on waking in the morning; it is at first slight, but intensifies till it may become most severe; indeed, almost unbearable. It affects one or both brows, and beginning at one spot, gradually extends, till it may involve the greater part of the head. The throbbing, stabbing, cutting, boring pain is increased by movement, noise, light, smells, or food. When the area of pain is limited, the complaint is termed *clavus*. As the pain subsides, or even during the whole attack, the patient may suffer dull or shooting pains in the eye of the affected side. There is much tenderness of the scalp during and after an attack.

Throughout the attack, the patient complains of nausea, which may be slight, but usually increases, and, when the pain is at its worst, ends in vomiting, which may be severe and prolonged, causing much prostration; yet occasionally vomiting affords relief.

Lasting a few hours, the whole day, or even two or three days, the attack generally ends in calm, refreshing sleep, but sometimes it gradually subsides or ends abruptly in vomiting, perspiration, or, more rarely, a copious flow of tears. The attack may be preceded and followed by very obstinate constipation or by diarrhœa, the liquid motions being in some instances pale, in others of a deep brown, mahogany color. Before and after the attack, there is often much dusky discoloration around the eyes.

It is now almost universally held that megrim is an affection of some part of the nervous centre. Dr. Liveing, to whose exhaustive work I am considerably indebted, considers that, in a typical case, the disturbance takes place first in the optic thalamus, and passes backwards and downwards, reaching to the nucleus of the vagus below; for, as he observes, in a typical seizure, the visual disorder is always the initial symptom, the headache the middle, and the vomiting last. Where morbid intellectual phenomena and disorder of speech occur, the affection radiates from the thalamus to the hemispheric ganglia, and, where emotional phenomena occur, to the mesocephale.

Though the affection is seated in the nervous centres, yet it must be recollected that the frequency and severity of the attacks both depend on peripheral causes, due to the stomach, intestines, liver, womb, etc. Even when the affection is strongly developed and the periodic attack recurs apparently spontaneously, the seizures may be rendered more frequent and severe by remote exciting causes; nay, in many cases, the affection may remain so slight that it lies dormant till roused into activity by some near or distant irritation, on removing which the seizures altogether cease.

The successful treatment of megrim depends less on change to be effected in the disordered nervous centres than on the removal of the existing cause. The treatment of megrim, therefore, falls under three heads:

1. The treatment of the central nervous affection;
2. The removal or prevention of exciting causes;
3. The treatment of the paroxysm.

Many remedies act in a two-fold or even three-fold way. Thus bromide of potassium is often extremely serviceable in two ways. It is very useful in those cases where the seizure is due to uterine disturbance, as in menorrhagia and dysmenorrhœa. Sometimes the attacks are more severe and frequent, arising from the exhausted state of the nervous system. Perhaps, from over-long town residence, or from mental troubles, the patient becomes irritable, depressed, nervous, excitable, with broken sleep, harassed by dreams. The ensuing general depression increases the headache. Now, bromide of potassium soothes the patient, and, by promoting refreshing sleep, strengthens the nervous system, and thus lessens the frequency and severity of the headaches. Bromide of potassium, moreover, is serviceable in the paroxysm itself, for it may produce several hours' sleep, from which the patient awakes free from headache.

The pain of megrim is situated in the fifth nerve; and, remembering how closely megrim is allied to neuralgia, and how useful hydrate of croton-chloral is in facial neuralgia, I have been induced to try this remedy for the seizures of megrim, and have found it useful in cases of which the following may be taken as a type:

A woman has been subject for years to nervous sick-headache; then, owing to some great trouble, or to excitement, fatigue, or flooding, or prolonged suckling, or most frequently at the change of life, the headache becomes much more severe. The headache is continuous for weeks, perhaps months, but is intensified greatly by fatigue, excitement, or at the catamenial period. If not actually continuous, the headache comes on daily, lasting, perhaps, for many hours, or several attacks may each day occur. The pain is often intense, and whereas, previously to the worst form of headache, the pain was probably limited to one bone, it now affects both, and perhaps the greater part of the head. The skin is generally very tender. There is also a sensation of bewilderment, or, as some term it, a stupid headache, and the patient often says she feels as if she should "go out of her mind." The sight may be dim, especially during the exacerbations of pain. Some patients of this class are very excitable and irritable, and are upset with the slightest noise. Nausea and even severe vomiting may occur with each exacerbation of the pain. Five grains of croton-chloral every three hours, or even oftener, will give in most cases considerable relief.—I need hardly say, that the drug does not entirely free the patient from her attacks; but, in one or two days, the pain ceases to be continuous, then the attacks recur, but only once or twice a week, the interval gradually extending till an onset occurs only every week, then about every fortnight, or even longer, till the illness assumes its old type and periodicity. In some cases, a week's treatment suffices to bring back the headache to its original type of an attack once in three or four weeks. Then the croton-chloral appears to be far less serviceable, manifesting but slight effect on the periodical attacks. In many cases of ordinary periodical headache, the patients say that, in the milder forms, the drug distinctly lessens the severity and duration, but in the severer forms it is without effect, even when sickness is absent. In those cases accompanied by severe vomiting and retching, croton-chloral is useless, being speedily rejected.

Croton-chloral, I have found, will relieve the slight attacks experienced by some delicate and nervous women after any slight fatigue or excitement.

In the continuous sick-headache just described, as the pain grows better so the cutaneous tenderness disappears. It seems to me that, in many instances, two kinds of headache coëxist, one sometimes predominating, sometimes the other. One appears due to affection of the cutaneous nerves, and is generally accompanied by tenderness. Patients describe the other as a "stupid headache," "a feeling of bewilderment," "a bewildering headache." After the dispersion of the first form by croton-chloral, this stupid headache often continues, but may ordinarily be relieved by bromide of potassium. Indeed, in many cases, I have found it useful to combine these remedies.—*British Medical Journal.*

